# Which Traits Make Weeds More Successful in Maize Crops? Insights from a Three-Decade Monitoring in France

**DOI:** 10.3390/plants9010040

**Published:** 2019-12-25

**Authors:** Guillaume Fried, Bruno Chauvel, François Munoz, Xavier Reboud

**Affiliations:** 1 Unité Entomologie et Plantes Invasives, Laboratoire de la Santé des Végétaux, Anses, 34988 Montferrier-sur-Lez, France; 2 Agroécologie, AgroSup Dijon, INRAE, Univ. Bourgogne, Univ. Bourgogne Franche-Comté, 21000 Dijon, France; bruno.chauvel@inra.fr (B.C.); xavier.reboud@inra.fr (X.R.); 3 Laboratoire d’Ecologie Alpine, Université Grenoble-Alpes, 38000 Grenoble, France; fmunoz@univ-grenoble-alpes.fr

**Keywords:** trait-based approach, diachronic study, abundance-occupancy relationship, weeds, *Zea mays*, specialist-generalist

## Abstract

A major aim in invasion biology is identifying traits distinguishing alien invasive and alien non-invasive plants. Surprisingly, this approach has been, so far, poorly used to understand why some arable weeds are abundant and widespread while others are rare and narrowly distributed. In the present study, we focused on the characteristics of successful weeds occurring in maize fields, one of the most important crops worldwide. Two national weed surveys conducted in France were used to identify increasing and decreasing species based on 175 and 484 surveyed fields in the 1970s and the 2000s, respectively. Weed trait values related to regional frequency, local abundance, and specialization to maize were identified with phylogenetic generalized least-squares (PGLS). We found a positive relationship between regional frequency and local abundance, i.e., the most widespread weeds were also locally more abundant. We highlighted that weeds with the C4 photosynthetic pathway and summer emergence were more abundant, more frequent, and more specialized to maize crops. More generally, we highlighted two successful strategies: On the one hand, traits related to a general weediness syndrome with rapid resource acquisition (high SLA and Ellenberg-N) and high colonization capacity (seed longevity, fecundity, and wind dispersal); on the other hand, traits related to specific adaptation to spring cultivation (thermophilous species with summer emergence, late flowering, and C4 photosynthetic pathway). Deviations from the abundancy–frequency relationships also indicated that species of the Panicoideae sub-family, species with Triazine-resistant populations, and neophyte species were more abundant than expected by their regional frequency. To some extent, it is therefore possible to predict which species can be troublesome in maize crops and use this information in weed risk assessment tools to prevent new introductions or favor early detection and eradication. This study showed how tools developed in functional and macro-ecology can be used to improve our understanding of weed ecology and to develop more preventive management strategies.

## 1. Introduction

Invasion ecology has a long tradition of searching for traits allowing to distinguish alien invasive and alien non-invasive plants [1,2]. The idea of the existence of specific characteristics related to invasiveness has its roots in the works of Baker [3,4] who published a list of trait attributes that an ideal weed should possess to rapidly spread and occupy a new area or previously vacant ecological niche. Surprisingly, such approach has been so far poorly extended and used to understand why some arable weeds are regionally frequent or locally abundant in crop fields while other are not [5,6,7]. A long-awaited result is to identify a typical ecological profile of weeds most impacting crop production, and to define management practices that limit them in the framework of an agroecological weed control approach [8]. Lososová, Chytry, and Kuhn [5] found that widespread arable weeds in cultivated fields of Czech Republic, mostly with winter cereals, were those flowering in pre-spring and early spring, adapted to low temperatures, relatively shade tolerant, and with high nutrient requirements. Fried, Chauvel, and Reboud [6] found that weeds that have increased in frequency in sunflower crops between the 1970s and the 2000s in France differed from decreasing weeds by high nutrient and light requirements, a lower sensitivity to sunflower herbicides, and a summer life cycle.

In these studies, the success of weed species have been related to trait values and strategies driving species frequency regionally. However, there are at least three facets of commonness [9] including (i) regional frequency, (ii) local abundance, and (iii) ecological specialization. Regional frequency tells us how widespread a weed is at the regional scale in the available habitat. One would expect that the regional frequency of a weed in a given crop reflects its ability to establish in many fields under the particular environmental conditions and specific management practices of that crop, either by emerging from the seed bank (temporal dispersion), or by successfully colonizing the field (spatial dispersion). Nevertheless, temporal or spatial dispersal can maintain weed populations despite non-optimal adaptation to the local management practices [10]. For instance, source-sink dynamics can maintain a high frequency of a weed present in other crops or in adjacent habitats, through spatial mass effect [11]. Then, regional frequency alone does not represent weed success well. In addition, regional frequency is based on the simple presence of a species (be it locally rare or abundant) and does not represent well the actual threat that a weed can represent locally, because the threat depends on local abundance and biomass.

Therefore, local abundance of a weed in a given crop is probably a better measure of its potential impact on yield, as well as a better measure of its success that can be more directly related to the local conditions created by the crop and the associated management practices. In a niche-based perspective, species should be most abundant under optimal niche conditions [12], and abundant weeds in a given crop should be those that possess trait values [13] best adapted to cope with the filtering by the specific management practices of that crop [14].

As a third facet of commonness, ecological specialization represents the extent to which a species occurs only in specific conditions (e.g., one crop type in our case) or is able to occupy a wide range of environmental conditions [15]. Ecological specialization of weeds can be measured over a gradient from generalist to specialist by relative abundance, i.e., the ratio between abundance in the habitat of interest (a given crop type) and abundance in other habitats (the other crop types) [16]. Compared to the two previous indices, ecological specialization is relative to a broad environmental context (i.e., frequency of occurrence or abundance in other conditions, other crops, or habitats). Dominance of weeds specialized to one crop type can reveal the repeated use of the same management practices and the too-frequent return of a crop in a crop sequence as shown with Brassicaceae weeds in fields with frequent oilseed rape crops [17]. Finally, in habitats that undergo dramatic changes in ecological conditions over time, such as arable lands where new practices are regularly adopted (weeding, new crop in the rotation, or soil preparation), another dimension of commonness that is useful to assess would be the status of increase or decrease over time (in terms of regional frequency or local abundance). There are a large number of studies that have shown that following changes in environmental conditions (e.g., eutrophication, herbivory pressure), it is possible to discriminate between trait values associated with “winner” species adapted to change, and traits of “loser” species disadvantaged by the new conditions [18,19].

In the present study, we focused on the characteristics of successful weeds occurring in maize fields. Maize (*Zea mays* L.) has become the first cereal grown in the world in terms of production tonnage. With 3.2 million ha (10% of the Utilized Agricultural Land), maize is the second most cultivated crop in France behind winter wheat. The area cultivated with maize have strongly increased from 450,000 ha in 1955 to 2,837,000 ha in 1975, and 3,186,000 ha in 2010. In France, maize is included in different cropping systems, more or less intensive with different input levels (irrigation) in relation to its major use as silage for cattle or seed production. These cropping systems vary from mono-cropping to crop sequences including temporary meadows, which potentially entail different types of weed communities [20]. Our hypothesis is that between the 1970s and the 2000s, the increasing maize cultivation has led to more frequent return of maize in the field and more frequent applications of a specific set of common management practices. We expect that these new conditions have filtered weed species with a suitable set of traits adapted to maize field selecting conditions.

Our study asks more precisely: (i) Which species have increased in frequency or in abundance in maize fields since the 1970s? (ii) Are intraspecific changes in regional frequency correlated to changes in local abundance of weeds in maize fields? (iii) At the interspecific level, are regional frequency and local abundance of weeds correlated for a given period, i.e., in the 2000s? (iv) To what extent are traits or syndrome of traits associated to regional frequency, local abundance, specialization to maize, and changes in the status of the species (decreasing, stable, increasing) between the 1970s and the 2000s?

## 2. Results

### 2.1. Changes in Weed Species Status

The assessment of changes in weed species status was based on comparing the regional frequency and local abundance in 175 fields surveyed in the 1970s and 484 fields surveyed in the 2000s. The 10 most frequent weeds remained very stable with eight species in common between the two periods, especially the top five species including *Chenopodium album*, *Echinochloa crus-galli*, *Persicaria maculosa*, *P. lapathifolia*, and *Amaranthus retroflexus*. Among common species already recorded in the 1970s, *Solanum nigrum* and *Mercurialis annua* were the only weeds significantly increasing in frequency, by +13% and +5%, respectively. Nine additional species increased and entered the top 30 most common species in maize, including six Dicotyledon weeds (e.g., *Calystegia sepium*, *Sonchus asper*, *Senecio vulgaris*, *Datura stramonium*) and three grasses (*Poa annua*, *Setaria verticillata*, *Lolium multiflorum*), ranging from +3% to +10% (Table 1). Seventeen species showed stable frequency over the studied period but their local abundance often decreased, except *Chenopodium album*, *Mercurialis annua*, *Stellaria media*, *Setaria pumila*, *Cynodon dactylon*, and *Alopecurus myosuroides*, which showed comparable level of abundance in the 1970s and the 2000s. Ten Dicotyledon weeds and two grasses decreased, although some species remained amongst the most common weeds (*Persicaria* spp., *Amaranthus retroflexus*, *Digitaria sanguinalis*), while some have now become relatively rare weeds in maize (*Fumaria officinalis*, *Raphanus raphanistrum*, *Spergula arvensis*, *Elytrigia repens*). Species that have developed resistant populations to triazine during the 1980s had variable trends: decrease (*Persicaria* spp.), increase (*Solanum nigrum*, *Senecio vulgaris*, or *Sonchus asper*) despite the withdrawal of this herbicide family, or remain stable (*C. album*, *E. crus-galli*).

Overall, there was a positive relationship between changes in regional frequency and changes in local mean abundance between the 1970s and the 2000s (phylogenetic generalized least-squares (PGLS), adjusted R^2^ = 0.371, F = 6.885, *p* = 0.028), i.e., species that increased (decreased) in frequency also increased (decreased) in abundance. Among species increasing since the 1970s, *Poa annua* and *Sonchus asper* showed a continuous increasing trend during the 2000s. *Persicaria* spp. and *Cynodon dactylon* also showed an increasing trend in recent years. On the other hand, *Alopecurus myosuroides* and *Stellaria media*, which were stable since the 1970s, and *Lamium purpureum* showed a decreasing trend. It was not possible to detect any consistent trend in the 2000s for other species (Table 1).

### 2.2. Regional Frequency, Local Abundance, and Specificity to Maize Crops

**Figure 1:**
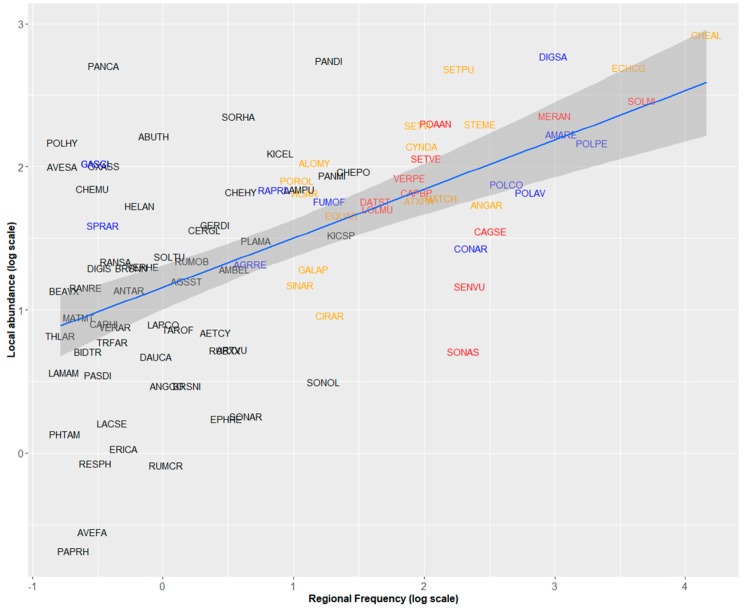
Relationships between regional frequency and local abundance of 95 arable weeds in maize fields recorded during the 2000 survey (phylogenetic generalized least-squares (PGLS) analysis, *F* = 42.61, Adj-R^2^ = 0.307, *p* < 0.001). Species names are abbreviated by EPPO Codes (https://gd.eppo.int/). Red: Increasing species, blue: Decreasing species, orange: Stable species. Black: Species for which the status cannot be determined.

In the 2000s, regional frequency of weeds in maize fields was positively related to their mean local abundance (PGLS analysis, *F* = 42.61, Adj-R^2^ = 0.307, *p* < 0.001, Figure 1). *Chenopodium album* was by far the most frequent and abundant weed in maize (present in nearly 60% of the surveyed fields, with a mean of 13.9 individuals/m^2^, Table 1, Figure 1). Six species displayed frequencies of occurrence between 20% and 40% including two grasses, *Echinochloa crus-galli* and *Digitaria sanguinalis* with higher abundances than Dicotyledon species with similar frequencies (Figure 1). Some weeds were locally abundant but regionally rare or moderately frequent (*Panicum capillare*, *Abutilon theophrasti*, *Sorghum halepense*, *Panicum dichotomiflorum*). Conversely, *Senecio vulgaris* or *Sonchus* spp. were regionally frequent but locally rare.

**Figure 2:**
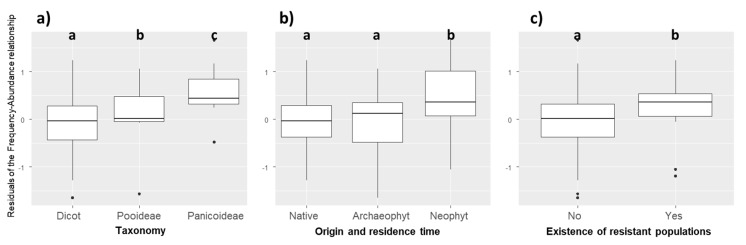
Distribution of the residuals of the frequency–abundance relationships based on (**a**) taxonomy (Kruskal–Wallis χ^2^ = 12.854, *p* = 0.002); (**b**) origin and residence time (Kruskal–Wallis χ^2^ = 6.534, *p* = 0.038); (**c**) existence of resistant populations (Kruskal–Wallis χ^2^ = 4.342, *p* = 0.037). Different letters indicate significant differences between groups (*p* < 0.05) based on post-hoc Dunn tests.

The residuals of the PGLS model were not independent of phylogeny (λ = 0.697). Compared to other species, weed species of the Poaceae family were more abundant than expected by their regional frequency (Kruskal–Wallis χ^2^ = 10.604, *p* = 0.001). More precisely, the residuals of weed species of the Panicoideae subfamily were also higher than those of Dicotyledon and Pooideae species (Figure 2a, Kruskal–Wallis χ^2^ = 12.854, *p* = 0.002). Neophyte species (i.e., alien plants introduced after 1500) had higher residuals than native and archaeophyte species (introduced before 1500) (Kruskal–Wallis χ^2^ = 6.534, *p* = 0.038, Figure 2b). Finally, weeds with known developed herbicide resistant populations (in France but not necessarily in the surveyed fields) had higher residuals (Kruskal–Wallis χ^2^ = 4.342, *p* = 0.037), i.e., their mean local abundance was higher than expected by their regional frequency (Figure 2c).

### 2.3. Traits Related to Success in Maize Crops

In order to understand the determinants of weed success in maize, we tested the relationship between 13 biological and ecological characteristics and the change in status between 1970 and 2000, local abundance, regional frequency, and degree of specialization in maize cultivation in the 2000s. No biological traits could be related to species status changes in frequency or abundance between the 1970s and the 2000s, except Ellenberg-N (PGLS, F = 4.658, *t*-value = 2.138, *p* = 0.036), which was higher for species increasing in local abundance and Elleberg-L (PGLS, *t*-value = 2.194, F = 4.813, *p* = 0.032) and Ellenberg-T (PGLS, F = 4.813, *p* = 0.032), which was higher for species increasing in regional frequency.

The PGLS models explaining variation in regional frequency, local abundance, and fidelity showed that life forms, photosynthetic pathways, SLA, emergence date, flowering onset, flowering duration, fecundity, seed longevity, Ellenberg-N, and Ellenberg-T were significant predictors of weed performance in maize whereas the other traits were not significantly related to any facet of performance (Table 2). Weed species with C4 photosynthetic pathway and summer emergence were more frequent, more abundant, and more specific to maize crop fields (Table 2). Species with higher seed longevity, higher temperature requirement, longer flowering duration, and ability to emerge all-year-round or from spring to summer were more frequent, while hemicryptophytes were less frequent (Table 2). Weed species with high SLA values and a spring-summer emergence were more abundant (Table 2). Finally, hemicryptophytes, species with high fecundity, and species emerging in spring or from spring to summer and with late flowering onset, were more specific to maize (Table 2).

### 2.4. Trait Syndromes Associated with Success in Maize

**Figure 3:**
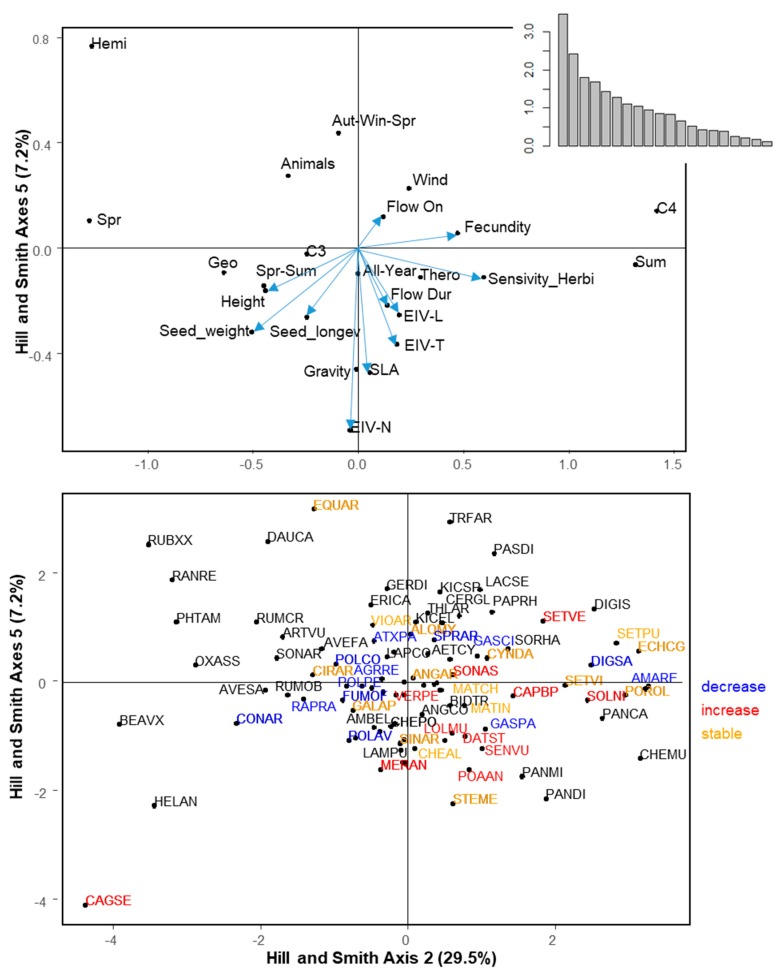
Hill and Smith analysis axes 2 and 5. These two axes were displayed because regionally frequent and locally abundant species are positively correlated to Axis 2 and negatively correlated to Axis 5. Top panel displays species traits: Continuous traits are represented by a vector and attributes of qualitative traits are represented by a black dot. Bottom panel displays species. Species names are abbreviated by EPPO Codes (https://gd.eppo.int/). Red: Increasing species, blue: Decreasing species, orange: Stable species. black: Species for which the status cannot be determined. The position of the species is represented by a black dot. For the sake of readability, not all species are represented by a label. When two species overlapped, the most frequent one is represented.

The first six axes of the Hill and Smith analysis explained 60.5% of the variation (Figure 3). Table 3 summarizes the correlation between traits and the first six axes of the Hill and Smith analysis. The PGLS models using Hill and Smith axes as explanatory variables showed that Axis 2 (positively related to C4 weeds, summer emergence, small seed weight, high fecundity, and wind dispersal) and Axis 5 (negatively related to SLA and Ellenberg-N) were positively and negatively correlated to regional frequency, respectively (Table 4). The same two axes (2 and 5) were significant explanatory variables for local abundance with the same direction (Table 4). Finally, specificity to maize showed a significant positive relationship to Axis 1 (tall geophytes, C4 weeds, with high light and temperature requirements, emerging in summer with a late flowering period) and axis 3 (hemicryptophytes with spring emergence, large flowering duration, and high seed longevity, Table 4). Changes in regional frequency between the 1970s and the 2000s could not be related to any axis of the Hill and Smith analysis, while changes in local abundance was negatively correlated to axis 5 (PGLS, *t*-value = −2.23, *p* = 0.030).

## 3. Discussion

The objective of this study was to identify weeds increasing in frequency and/or abundance in maize fields in France between the 1970s and the 2000s, and to determine which traits or trait syndromes were associated with success of arable weeds in this crop. While the ranking of the most common weed species in maize fields remains relatively stable between 1973 and 2010, nine species entered the top 30 species, revealing the colonization of thousands of maize fields in France by « new » weeds during recent decades. A significant 10% change in frequency at the scale of the whole maize area meant huge changes within the maize weed flora. We found that partly distinct trait values explained local abundance and specificity to maize on one hand (C4 species with spring–summer emergence), and regional frequency on the other hand (therophytes and geophytes with high seed longevity, large flowering duration, and ability to emerge all-year-round), while changes in regional frequency or local abundance between the 1970s and the 2000s were poorly associated with biological traits with only an increase of nutrient-demanding species with high light and temperature requirements. A multivariate analysis identified several axes of specialization gathering sets of traits, some of which were clearly related to a ruderal strategy [21], particularly an axis related to resource acquisition (SLA, Ellenberg-N), and an axis related to colonization capacity (seed weight, wind dispersal) while others reflect a more specific adaptation to spring cultivation (thermophilous species with summer emergence and C4 photosynthetic pathway).

### 3.1. Trends Since the 2000s

Given the intensification of agricultural practices since the 1970s and the more frequent return of maize in crop rotations, it is surprising that no biological trait was related to changes in species status, unlike what had been shown for sunflower over the same period [6]. The only feature significantly related to changes in species status highlighted an increase in the local abundance of nutrient-demanding species and species with a rapid acquisition trait syndrome (Axis 5) as well as an increase in regional frequency of light-demanding species with high requirement in temperature. These results suggest a strong response of weed communities to increased fertilization levels at a nationwide level [22]. Increased fertilization is a change in practice that has affected all crops and is therefore more likely to have a global effect compared to changes specific to one crop, which can be buffered by other crops of the crop succession. Moreover, two features characterizing maize cultivation can limit the potential filtering effect over time. First, the possibility to use maize as fodder allows the farmer to harvest the crop before a large proportion of the weed flora species have produced their seeds, which would reduce the selection of species mimicking maize life cycle. Second, maize, unlike other crops, can still be chemically weeded with a wide range of active substances, which can reduce trait selection to a dominant active substance. The absence of traits related to changes in the status, and the stability of the most frequent and abundant species in maize between 1973 and 2010 can also indicate that the main selective pressures exerted by the crop and management practices in maize had already sorted the species in the 1970s and have remained quite similar since then. Similarly, in a study from Spain, little changes were observed between 1989 and 2009 in maize crops, with predominance of alien grasses and C4 species mainly due to mono-cropping [23]. In Italy, a similar diachronic study, yet spanning over a longer period from 1964 to 2017, showed that increasing species were mostly neophytes, C4 species, monocotyledons, and geophytes [24]. When considering traits related to frequency, abundance, and specificity to maize in the 2000s (Table 2), we found consistent results with several recent studies on maize weed communities [23,24,25] showing that neophytes, panicoids, C4 species, perennials, or summer annuals are more successful or specific to maize fields. In the early 2000s, withdrawal of atrazine was mentioned as a major problem with a high risk of weed flora change [26]. In other studies, an increase of weed diversity was mentioned [27]. The data used in this study do not show that there has been any significant change certainly due to alternatives in chemical weed control.

### 3.2. Weediness Traits

Certain traits related to high frequency or abundance relates to generic features of weediness. These traits can be divided in two categories. The first category of frequent and abundant weeds in maize shares rapid resources acquisition capacities through high SLA and high Ellenberg-N values. This is consistent with a recent study that showed that weeds were distinguished from other herbaceous species in open habitats by high values of SLA and Ellenberg-N, and that among crop weeds, those most specific to the cultivated environment also had high values of these indices [28]. The second category of frequent and/or abundant weeds have a strong colonization capacity with a low seed weight combined with a high fecundity. These features are adapted to both spatial and temporal dispersal. High seed production is advantageous in intensive farming conditions as the resulting high seed bank can compensate the mortality due to (chemical) weeding [29].

### 3.3. Filtering of Crop Mimicking Traits

Our results support the crop mimicry hypothesis that species most successful in a crop are those that most closely resemble that crop [23,30,31]. First, the most abundant and specific weeds to maize are species that germinate in spring-summer and that have a late flowering, i.e., that have the same life cycle as maize. Synchronicity between weed emergence and crop seeding date is probably the most consistent feature explaining species success in a crop [6,17,32] or differences in species assemblage between crops [10,33]. It can be pointed out that this trait influences local abundance and maize specificity but not regional frequency, which confirms our hypothesis that traits related to abundance and specialization are more related to specific local environmental conditions of the crop, while traits related to regional frequency can be more related to overall success in cultivated fields, e.g., with broader range of flowering period [15].

Second, abundant weeds specific to maize more often had a C4 photosynthetic system (such as maize), well adapted to high temperatures during the summer cycle of the crop where greater photosynthetic efficiency makes them more competitive than C3 weeds. The trait syndromes analysis also indicated that successful weed species in maize had high temperature and light requirements, which corresponds well to conditions where C4 plants have a competitive advantage over C3 plants.

Finally, the residuals of the PGLS analyses between frequency and abundance were not independent from phylogeny. This means that the variance-covariance structure considered “neutral” in the PGLS analysis between regional frequency and local abundance is invalidated, and that there are lineages with specific behaviors that this structure cannot take into account. Plotting residuals showed that weeds of the Panicoideae subfamily (same subfamily as maize) were more abundant than expected by their frequency (Figure 2). Considering that phylogenetically related species share similar trait values, this reinforces the hypothesis of mimicry with the crop species including its pattern of herbicide tolerance.

### 3.4. Significance of Frequency–Abundance Relationships for Weed Science

The abundance–frequency relationship is a classic pattern largely studied in macroecology [34]. Our study shows that it can be very informative in the case of arable weeds (Figure 1). Interesting cases are species that deviate from the relationship, i.e., species that have either a higher or a lower abundance than expected by their regional frequency (Figure 2). Among species with higher than expected abundance, we found mainly potentially troublesome weed species. First, we have shown that this is the case of panicoid grass weeds. Their higher than expected abundance can result from difficulty to control species botanically close to the crop. Second, we found this pattern for invasive neophytes (*Panicum capillare*, *Abutilon theophrasti*, *Sorghum halepense*, *Panicum dichotomiflorum*). These species form locally dense stands but as they have been introduced more recently than native or archaeophyte weeds, they have not yet reached the limits of their potential distribution area in France, which explains why they do not fit the global frequency–abundance relationship. Finally, weeds that have developed resistant populations to triazines (*Digitaria sanguinalis*, *Solanum nigrum*, *Setaria* spp.) also had higher abundance than expected. In this case, it is difficult to know if these species are more abundant than expected in the 2000 surveys because they have developed resistant populations during the 1980s or if they have become resistant because they were already abundant and therefore more likely to select resistant mutants. Both aspects have probably played a role.

## 4. Materials and Methods

### 4.1. Weed Surveys

**Figure 4:**
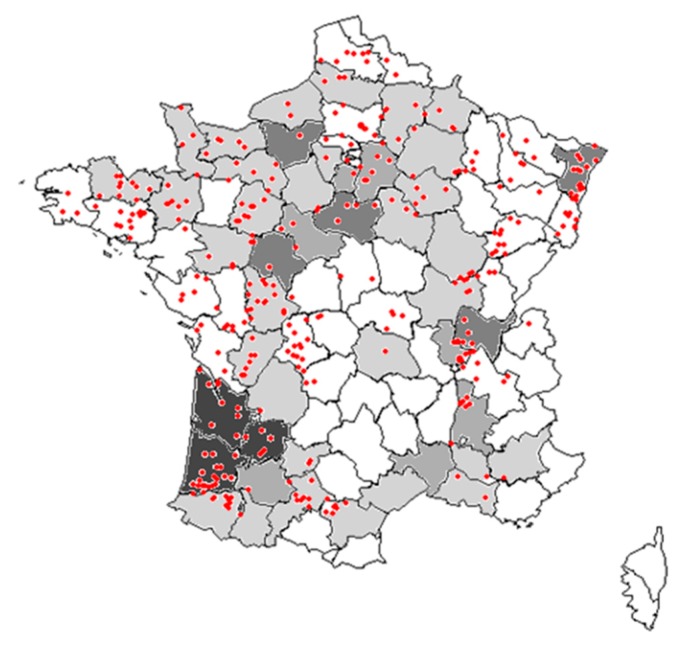
Map of surveyed fields in France. Departments in grey were those surveyed in the 1970s (with darker grey indicated more plots in this department; in white no survey). The red points indicate the locations of plots surveyed in the 2000s.

Local abundances, regional frequency of occurrence, and long-term changes in frequency and abundance of common weed species in maize crops were assessed based on two national weed surveys. The first survey was conducted between 1973 and 1976 [35] and sampled a total of 2170 fields across France, 175 of which in maize crops in five specific areas of France (Figure 4). For this first survey, only the frequency of occurrence and mean density of the 29 most frequent weeds were available. The second survey was conducted at least 25 years latter between 2002 and 2010 in the framework of the ‘Biovigilance Flore’ monitoring [36], which included 998 samples in 484 different fields with maize crop (out of 5382 samples).

For the 2000s, two plots were established in each field: Weeds were recorded in one control plot (C) of ~100–150 m^2^ (identical soil preparation and sowing practices, but no chemical or mechanical weeding, allowing the soil seedbank to express the potential weed species present in the field) and in one adjacent herbicides treated plot (T) of 2000 m^2^ (50 × 40 m), both located at least 20 m from field boundaries. A total of 38 different active ingredients were used over all plots in the 2000s, including nine applied in more than 10% of the fields: Nicosulfuron (38%), sulcotrione (27%), mesotrione (26%), aclonifen (24%), acetochlore (21%), atrazine (19%, only up to 2003), dicamba (16%), bromoxynil (16%), alachlore (11%). In each plot, species abundance was recorded using six cover abundance classes, adapted from Barralis [34], i.e., + = 1 individual/2000 m^2^; 1 = <1, 2 = 1–2, 3 = 3–20, 4 = 21–50, 5 = >50 individuals/m^2^. For the 1970s, weeds were only recorded in the control plots, but the weed sampling strategy was similar in the two surveys.

### 4.2. Measures of Regional Frequency, Mean Local Abundance, Specialization, and Status Changes

The frequency of occurrence (F) of a weed species was the number of fields where it occurred divided by the total number of sampled fields. Local mean abundance was calculated as the average density in sites where the species was present. Based on the 6-class abundance scale used in the fields, a local mean abundance (A) was computed as follows:

(1) $$A=\frac{\left[11.5\times n3+35.5\times n4+75.5\times n5+1.5\times \left(N-n3-n4-n5\right)\right]}{N}$$

where n3, n4, and n5 are the number of fields where the species was noted at scores 3, 4, and 5, respectively, and N is the total number of occurrences of the species [35]. The third index, related to ecological specialization, was based on a measure of fidelity to maize cultivation. Fidelity is the proportion of the individuals of species i that was found in maize relative to all individuals including those found in other crops, using the whole Biovigilance dataset [37].

We compared species regional frequency and local abundance between the two national weed surveys conducted in France in the 1970s and the 2000s based on the control plots only. In order to homogenize the sampling effort between the two surveys (175 vs. 484 fields), a bootstrap procedure was conducted on the 2000s dataset and adjusted to the smallest sampling size. Since we know the distribution of the number of fields per region in the 1970s (East: 26, South-West: 64, North-Parisian basin: 41, West: 26, South-East: 17), we used a stratified bootstrap procedure to conserve an equal distribution of field numbers across regions for the two surveys. From the bootstrap resampling of the 2000s dataset, we calculated a 95% confidence interval around the mean frequency and mean abundance of a given species. Significant change in species frequency or abundance between periods was observed when its frequency or abundance in the first survey was outside of the 95% bootstrap confidence interval of the frequency or abundance calculated from the second survey (Table 1).

To compare frequency and abundance of weed species within the 2000s period, we applied a similar bootstrap procedure keeping a similar distribution of samples across regions for each year (adjusted to the smallest regional sample size of the period 2002–2008). Trends in species frequency and abundance within the 2000s were then estimated by a Spearman rank correlation test between mean frequency (or mean abundance) and year (from 2002 to 2008).

### 4.3. Weed Traits and Phylogeny

Nine biological traits, one functional type, and three indices of ecological requirements were selected to identify response traits related to performance in maize (Table 5). The selected traits included the three traits of the leaf–height–seed (L-H-S) strategy scheme [38]. In the context of arable weed communities, specific leaf area (SLA, the ratio of leaf surface to leaf dry mass) has proved to be related to weed relative growth rate in spring [39]. Plant height determines the impact of competition for light between weeds and the crop [40]. Seed weight is the result of a trade-off between producing a few large seeds, with higher probability of a successful establishment, and producing many small seeds, with a low probability of establishing but greater dispersal [41]. In agriculture, it can also be subjected to strong selective pressure during crop seed sorting procedures [17]. Photosynthetic pathways (distinguishing C3 and C4 pathways) were also used because C4 plants are expected to be favored in C4 crops such as maize [42]. Traits associated with persistence in disturbed and ephemeral habitats, such as phenological traits (germination and flowering periods), the mode of species dispersal, as well as fecundity (seed production) and seed longevity estimation, were also included [43,44]. Four emergence dates were distinguished: i) Species that can germinate all year round; ii) species that germinate in autumn, spring, and summer; iii) species that germinate in spring only; iv) species that germinate in spring and summer; and v) species that germinate in summer only. The onset and duration of flowering was also considered as relevant information on the ability of species to complete their life cycle during maize cultivation. Three classes of seed dispersal were distinguished: By animals (epizoochory, endozoochory, or myrmecochory), by gravity, or by wind. Together with these eight traits, Raunkiaer’s life forms (therophytes: Th., geophytes: Geo., hemicryptophytes: Hcr.) were considered because this plant classification has been successfully used to illustrate the response of weeds to the level of soil disturbance by tillage systems [45]. Several indicator values proposed by Ellenberg et al. [46] could be directly related to agricultural management filtering or global change: The increasing level of fertilization supply is expected to favor nitrophilous competitive weeds (Ellenberg-N) [47] while row spacing and crop canopy height could influence the establishment of species based on their shade tolerance (Ellenberg-L). Change in the mean Ellenberg indicator for temperature requirements (Ellenberg-T) could indicate the influence of climate change [48] or shift towards spring-sown crops [24]. Finally, sensitivity to herbicides registered in maize crops in the 2000s was obtained from Mamarot and Rodriguez [49]. This index represents the average response of sensitive weed populations in maize and does not take resistant populations into account.

We also categorized Dicotyledon and grass weeds, Panicoideae, and other grasses (Pooideae) within the Poaceae family, as well as the existence of resistant populations to herbicides used in maize (only triazine-resistant plants in France; Darmency and Gasquez [56]). The distinction between weeds from the Panicoideae sub-family and other weeds is particularly important in maize, a grass crop that is hypothesized to favor closely related Panicoideae weeds under the crop mimicry hypothesis [30]. Plant species were classified into native, archaeophytes (i.e., alien introduced before 1500), and neophytes (alien introduced after 1500) using several French floras [51,57]. The units of the traits are given in Table 5. Missing values of traits were filled with predictive mean matching using the mice package (for nine species for seed weight and for two species for SLA).

### 4.4. Data Analysis

**Figure A1:**
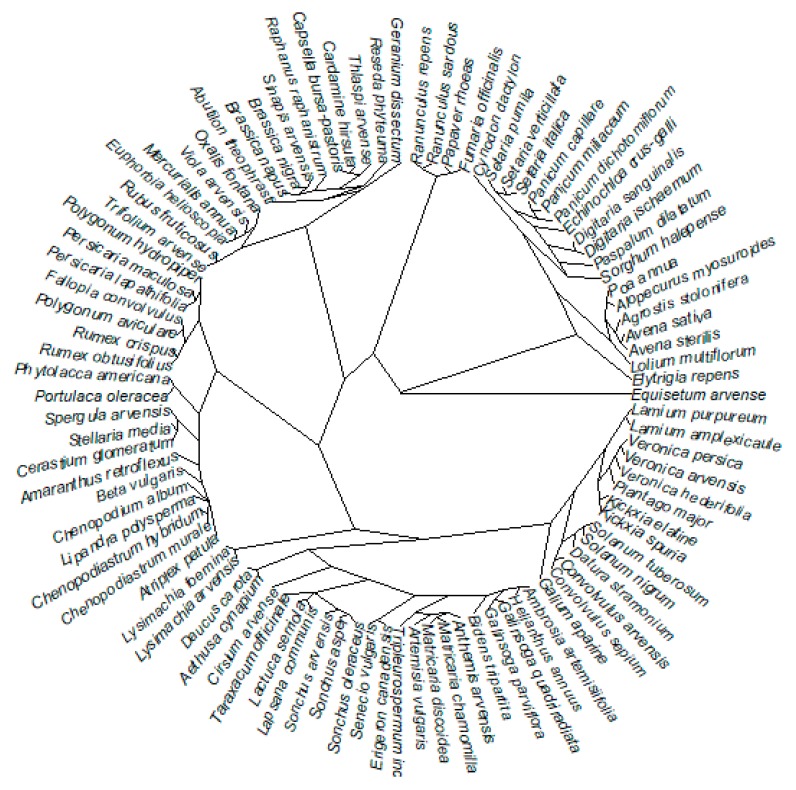
Phylogenetic tree of weed species found in maize in France.

To analyze the variation in species success in maize explained by traits while accounting for phylogeny, we developed phylogenetic generalized least-squares (PGLS) models with the PGLS function of the R package caper [58]. Because of phylogenetic relatedness, species did not represent independent data point for analyses. Some of the relationships identified between regional frequency and local abundance of species, and between indices of commonness and species attributes, could reflect phylogenetically related species are more similar due to their common evolutionary history [59]. Such phylogenetic non-independence could bias statistical tests assuming independence between individual species values. Therefore, we controlled for phylogenetic relatedness in our statistical analyses. The phylogeny of arable weed species of our dataset was generated with the function S. PhyloMaker provided by [60] with scenario “S3”, which derived from a dated and comprehensive megaphylogeny of spermaphytes (see Figure A1 in Appendix B).

For weed performance in the 2000s, response variables were regional frequency, local abundance, and specificity to maize crops. For changes in species status (*Ch.*) between the 1970s and the 2000s, the response variables correspond to the differences in regional frequency or local abundance between the 2000s and the 1970s. We used the following formula to achieve normality

(2) $$Ch.=\frac{S_{2000s}-S_{1970s}}{S_{2000s}+S_{1970s}}$$

where *S*_1970s_ is the regional frequency or local abundance in the 1970s and *S*_2000s_ is the regional frequency or local abundance in the 2000s.

Two complementary modelling approaches were used. In the first approach, each trait was used separately in PGLS models. This first approach is limited by the fact that it does not take into account the correlations that may exist between species traits. In order to take this correlation into account and to identify if species success was associated to a combination of particular traits or trait syndromes, the second approach was based on a multivariate analysis. The species–traits matrix (95 species x 15 traits) was first subjected to a Hill and Smith analysis (a multivariate analysis similar to principal component analysis allowing both quantitative and qualitative variables). The relative contributions of each trait to the decomposition of total inertia between axes were analyzed to interpret each axis as trait syndromes (Table 3). Then, the Hill and Smith axes (combination of traits) were used as explanatory variables in the PGLS models (Table 4). Plant height, seed weight, fecundity, and seed longevity were also log-transformed to make explanatory variables conform to normality. Local mean abundance and regional frequency were log-transformed prior to the analyses. PGLS residuals were inspected visually and based on Shapiro–Wilk normality test to detect trends that could bias estimates, but all models behaved properly.

In PGLS models, branch length transformations were applied to assess the most meaningful phylogenetic covariance structure in the model. Original branch lengths could be multiplied by a factor λ and/or elevated at a power of δ. Maximum likelihood estimates of λ and δ were calculated and compared to a situation where phylogenetic relatedness did not influence the relationship between commonness indices and biological traits (λ = 0), using likelihood ratio tests. If the difference was not significant, PGLS models were equivalent to standard GLS models without the influence of phylogenetic relatedness. If residuals were significantly influenced by phylogeny, we particularly compared the distribution of residuals according to species belonging to Poaceae or Panicoideae versus other species. These comparisons were based on Kruskal–Wallis tests and Dunn tests (for pairwise comparisons).

## Figures and Tables

**Table 1:** Regional frequency, local abundance, and status changes between the 1970s and 2000s and during the 2000s for the 40 most frequent weeds in the 2000s, and for four additional taxa that were amongst the 29 most frequent in the 1970s (the complete list of species observed in the 2000s survey is given in Table A1 in Appendix A).

Rank	Names ^1^	Regional Frequency (%)			Local Abundance (ind./m^2^)			Trend in the 2000s		
		2000s ^2^	1970s	Status ^3^	2000s ^2^	1970s	Status ^3^	Spearman rho	*p*-Value	Status ^3^
1	*Chenopodium album*	64.3 [57.7–70.3]	60.3	=	13.9 [11.2–16.6]	12.4	=	−0.250	0.594	=
2	***Solanum nigrum***	**39.1 [33.1–45.1]**	**26.5**	+	7.5 [5.5–9.6]	6.9	=	−0.143	0.783	=
3	*Echinochloa crus-galli*	35.5 [29.7–41.1]	38.0	=	8.4 [6.2–10.6]	12.9	−	0.357	0.444	=
4	***Persicaria maculata + P. lapathifolia***	26.7 [21.1–32.0]	35.1	−	**4.9 [3.4–6.6]**	**3.1**	**+**	**0.786**	**0.048**	**+**
5	*Amaranthus retroflexus*	21.1 (16.0–25.7]	26.7	−	3.6 [2.3–5.2]	5.8	−	0.071	0.906	=
6	***Mercurialis annua***	**20.1 [15.5–25.1]**	**15.4**	**+**	3.3 [2.1–4.7]	3.9	=	0.286	0.556	=
7	*Digitaria sanguinalis*	19.8 [15.4–24.6]	40.2	−	5.2 [3.4–7.1]	14.5	−	0.214	0.662	=
8	*Polygonum aviculare*	16.6 [12.6–21.1]	26.4	−	2.1 [1.4–3.1]	7.5	−	−0.357	0.444	=
9	*Fallopia convolvulus*	13.9 [9.7–18.3]	21.3	−	1.8 [1.0–2.6]	4.1	−	0.536	0.236	=
10	***Calystegia sepium***	**12.3 [8.0–16.6]**	**<2.3**	**N**	**1.7 [1.0–2.6]**	**?**	**N**	**0.750**	**0.066**	**(+)**
11	*Lysimachia arvensis*	12.0 [8.0–16.0]	13.2	=	1.1 [0.6–1.9]	2.9	−	0.571	0.200	=
12	*Stellaria media*	11.3 [7.4–15.4]	14.3	=	2.6 [1.4–4.0]	3.6	=	**−0.928**	**0.007**	**−**
13	*Convolvulus arvensis*	10.6 [6.9–14.3]	15.5	−	1.4 [0.8–2.2]	2.8	−	0.143	0.783	=
14	***Senecio vulgaris***	**10.5 [6.9–14.3]**	**<2.3**	**N**	**1.2 [0.9–1.7]**	**?**	**N**	**0.714**	**0.088**	**(+)**
15	***Sonchus asper***	**10.0 [6.8–13.7]**	**<2.3**	**N**	**1.2 [0.8–1.7]**	**?**	**N**	**0.893**	**0.012**	**+**
16	*Setaria pumila*	9.6 [6.3–16.1]	9.8	=	2.9 [1.4–4.5]	3.6	=	0.464	0.302	=
17	*Matricaria chamomilla + Tripleurospermum inodorum*	8.4 [5.1–12.0]	7.9	=	1.3 [0.7–2.1]	2.3	−	0.607	0.167	=
18	***Poa annua***	**8.1 [4.6–11.4]**	**<2.3**	**N**	**1.4 [0.6–2.5]**	**?**	**N**	**0.857**	**0.024**	**+**
19	***Setaria verticillata***	**7.5 [4.0–10.9]**	**<2.3**	**N**	0.9 [0.4–1.6]	?	?	**0.750**	**0.066**	**(+)**
20	***Cynodon dactylon***	7.2 [4.0–10.3]	5.2	=	1.5 [0.6–2.6]	1.3	=	**0.857**	**0.024**	**+**
21	*Atriplex patula*	7.1 [4.0–10.3]	10.3	=	1.0 [0.4–1.6]	2.8	−	0.429	0.353	=
22	*Setaria viridis*	7.1 [4.0–10.3]	10.3	=	1.3 [0.5–2.3]	4.2	−	0.286	0.556	=
23	***Capsella bursa-pastoris***	**7.0 [4.0–10.3]**	**<2.3**	**N**	**1.0 [0.5–1.8]**	**?**	**N**	−0.643	0.139	=
24	***Veronica persica***	**6.6 [3.4–9.7]**	**<2.3**	**N**	1.0 [0.4–1.8]	?	?	0.250	0.595	=
25	***Lolium multiflorum***	**5.2 [2.3–8.0]**	**<2.3**	**N**	0.7 [0.3–1.2]	?	?	−0.464	0.302	=
26	***Datura stramonium***	**5.1 [2.3–8.0]**	**<2.3**	**N**	1.1 [0.4–1.9]	?	?	−0.036	0.964	=
27	*Lipandra polyspermum*	4.3 [1.7–7.4]	<2.3	?	0.5 [0.2–1.0]	?	?	−0.071	0.906	=
28	*Equisetum arvense*	3.9 [1.7–6.3]	6.2	=	0.5 [0.2–1.1]	1.9	−	0.071	0.906	=
29	*Kickxia spuria*	3.9 [1.7–6.3]	<2.3	?	0.4 [0.2–0.7]	?	?	0.607	0.167	=
30	*Panicum miliaceum*	3.7 [1.7–6.3]	<2.3	?	0.7 [0.2–1.3]	?	?	−0.179	0.713	=
31	*Cirsium arvense*	3.6 [1.7–6.3]	6.3	=	0.5 [0.3–0.9]	1.6	−	−0.214	0.662	=
32	*Fumaria officinalis*	3.6 [1.7–6.3]	6.4	−	0.8 [0.3–1.4]	1.4	=	0.536	0.236	=
33	*Panicum dichotomiflorum*	3.6 [1.1–6.3]	<2.3	?	0.8 [0.2–1.6]	?	?	0.036	0.966	=
34	*Sonchus oleraceus*	3.4 [1.1–5.7]	<2.3	?	0.5 [0.3–0.9]	?	?	0.214	0.662	=
35	*Alopecurus myosuroides*	3.2 [1.1–5.1]	2.9	=	0.8 [0.2–1.5]	0.8	=	−0.821	0.034	−
36	*Galium aparine subsp. aparine*	3.2 [1.1–5.1]	4.6	=	0.4 [0.2–0.8]	1.1	−	0.500	0.267	=
37	*Viola arvensis*	2.9 [1.1–5.1]	4.6	=	0.4 [0.1–1.0]	1.4	−	−0.464	0.302	=
38	*Sinapis arvensis*	2.9 [1.1–5.1]	4.6	=	0.4 [0.2–0.8]	2.0	−	−0.428	0.354	=
39	*Lamium purpureum*	2.8 [1.1–5.1]	<2.3	?	0.4 [0.1–0.9]	?	?	−0.929	0.007	−
40	*Portulacca oleracea*	2.8 [1.1–5.1]	2.3	=	0.8 [0.1–1.6]	1.8	−	−0.429	0.353	=
42	*Raphanus raphanistrum*	2.3 [0.6–4.6]	15.6	−	0.4 [0.1–1.1]	3.0	−	0.643	0.139	=
44	*Elytrigia repens*	1.9 [0.6–4.0]	4.5	−	0.3 [0.1–0.8]	2.1	−	0.107	0.840	=
77	*Spergula arvensis*	0.6 [0.0–1.7]	6.9	−	0.1 [0.0–0.2]	2.9	−	−0.211	0.669	=
81	*Galinsoga quadriradiata + G. parviflora*	0.6 [0.0–1.7]	2.3	−	0.1 [0.0–0.3]	0.4	−	0.556	0.256	=

^1^ Species in bold are species significantly increasing in frequency or in abundance between the 1970s and the 2000s or showing an increasing trend in the 2000s. ^2^ Values between brackets corresponds to the 95¨% confidence interval around the mean frequency or abundance based on the 2000 bootstrap resampling. ^3^ “N”: new species not recorded in the 1970s survey (with a frequency < 2.3% in the 1970s), “+”: increasing species, “=”: stable species, “−”: decreasing species, “?”: “species for which no status can be determined”, “(+)”: species showing a non-significant increasing trend during the 2000s (0.05 < *p* < 0.10).

**Table 2:** Results of PGLS models with the estimate, standard error, *t*-value, and *p*-value for each trait and for each model. *p* < 0.05, * *p* < 0.01, ** *p* < 0.001. Bold character indicates traits significantly related to performance in maize.

	Regional Frequency				Local Abundance				Specificity			
	Estimate	Std. Err.	*t*-Value	*p*-Value	Estimate	Std. Err.	*t*-Value	*p*-Value	Estimate	Std. Err.	*t*-Value	*p*-Value
**Hemicryptophytes**	**−1.247**	**0.523**	**−2.384**	0.019 *	−0.447	0.306	−1.463	0.147	**23.945**	**10.214**	**1.251**	0.021 *
Therophytes	0.238	0.412	0.579	0.564	0.209	0.240	0.873	0.385	0.793	8.000	0.099	0.921
**C4**	**0.760**	**0.358**	**2.124**	0.036 *	**0.854**	**0.205**	**4.163**	<0.001 ***	**34.053**	**6.734**	**5.057**	<0.001 ***
Plant Height	0.280	0.206	1.362	0.177	−0.126	0.124	−1.016	0.312	−2.583	4.238	−0.610	0.544
Seed weight	0.011	0.087	0.123	0.903	−0.005	0.050	−0.091	0.928	−2.598	1.621	−1.602	0.113
**SLA**	0.005	0.013	0.379	0.706	**0.016**	**0.007**	**2.248**	0.027 *	−0.083	0.236	−0.351	0.727
**All-year-round**	**0.851**	**0.379**	**2.242**	0.027 *	0.248	0.218	1.141	0.257	9.493	7.031	1.350	0.180
**Spring**	0.398	0.503	0.790	0.431	0.225	0.289	0.781	0.437	**22.534**	**9.322**	**2.417**	0.018 *
**Spring & Summer**	**1.284**	**0.393**	**3.270**	0.002 **	**0.654**	**0.225**	**2.903**	0.005 **	**26.125**	**7.277**	**3.590**	0.001 **
**Summer**	**1.310**	**0.417**	**3.141**	0.002 **	**1.097**	**0.239**	**4.581**	**<0.001**	**43.553**	**7.730**	**5.634**	<0.001 ***
**Flow. onset**	0.008	0.073	0.104	0.917	−0.038	0.046	−0.894	0.374	**4.045**	**1.398**	**2.893**	0.005 **
**Flow. duration**	**0.133**	**0.055**	**2.409**	0.018 *	0.046	0.032	1.414	0.161	−0.838	1.076	−0.779	0.438
**Fecundity**	0.036	0.075	0.485	0.628	0.019	0.044	0.438	0.664	**3.361**	**1.432**	**2.327**	0.021 *
**Seed longevity**	**0.272**	**0.122**	**2.239**	0.028 *	0.094	0.075	1.265	0.209	2.819	2.479	1.137	0.258
Gravity	0.201	0.335	0.599	0.551	0.162	0.201	0.805	0.423	2.467	6.856	0.360	0.720
Wind-dispersal	0.366	0.327	1.117	0.267	0.148	0.200	0.743	0.460	2.008	6.843	0.294	0.770
Ellenberg-N	0.091	0.088	1.038	0.302	0.100	0.051	1.946	0.055	−2.082	1.766	−1.179	0.242
Ellenberg-L	−0.109	0.146	−0.749	0.456	−0.013	0.085	−0.150	0.881	2.268	2.759	0.822	0.413
**Ellenberg-T**	**0.446**	**0.141**	**3.152**	0.002 **	0.106	0.085	1.250	0.215	2.732	2.770	0.986	0.327
Sensitivity to Maize herbicides	0.069	0.119	0.579	0.564	0.152	0.067	1.263	0.126	−3.568	2.302	−1.550	0.125

**Table 3:** Relative contribution (%) of trait (modalities) to the Hill and Smith analysis (HAS) axes. Blue cells indicate positive relationships while red cells indicate negative relationships, the darker the color the stronger the contribution.

Traits	Axis1	Axis2	Axis3	Axis4	Axis5	Axis6
**Geophyte**	17.30	−4.83	−2.16	32.84	−0.10	−0.12
**Hemicryptophyte**	2.86	−23.21	28.20	−5.56	8.52	7.94
**Therophyte**	−19.00	29.08	−9.69	−5.35	−4.25	−3.86
**Plant Height**	31.37	−19.29	−4.90	−0.15	−2.67	4.98
**Seed weight**	3.67	−25.66	−16.47	−11.30	−10.27	−0.03
**SLA**	−23.63	0.29	−2.34	0.40	−22.55	0.83
**Autumn-Winter-Spring**	−24.66	0.00	5.41	6.44	−0.42	−0.06
**All-year-round**	−8.25	−0.16	−32.73	−5.71	3.61	0.07
**Spring**	0.08	−17.00	11.47	−21.01	0.11	−5.46
**Spring-Summer**	8.70	−6.85	−0.24	35.25	−0.72	1.30
**Summer**	25.28	40.32	0.36	−15.34	−0.09	0.26
**Flowering Onset**	51.05	1.40	0.00	0.03	1.40	0.80
**Flowering Duration**	−35.68	1.85	19.92	1.08	−4.76	−0.04
**Fecundity**	3.67	21.96	2.93	−5.82	0.30	25.12
**Seed longevity**	−0.10	−6.15	45.85	−1.85	−7.04	−3.88
**Animals**	−1.35	−4.02	−0.24	−4.98	2.72	−14.50
**Gravity**	1.35	−0.01	−1.90	−12.07	−11.34	−1.02
**Wind**	−0.01	3.61	3.19	29.23	3.24	19.58
**Ellenberg-N**	−0.22	−0.15	0.08	−2.19	−48.51	25.78
**Ellenberg-L**	37.11	3.78	−0.76	0.31	−6.68	−14.20
**Ellenberg-T**	37.42	3.31	8.43	8.77	−13.62	−10.37
**C3**	−26.31	−34.62	−1.18	5.31	−0.34	0.01
**C4**	26.31	34.62	1.18	−5.31	0.34	−0.01
**Sensitivity to Herbicides**	−21.30	34.98	−2.58	0.12	−1.25	−3.56

**Table 4:** PGLS models on Hill and Smith (H&S) axes. HS 1 to 6 refers to Hill and Smith axes. Est.: Estimates, S.E.: Standard Error, *t* val: *t*-values, *p* val: *p* values. Bold values indicate Hill and Smith (HS) axes significantly related to performance in maize.

	Regional Frequency				Local Abundance				Specificity to Maize			
	Est.	S. E.	*t* val	*p* val	Est.	S. E.	*t* val	*p* val	Est.	S. E.	*t* val	*p* val
**HS 1**	0.056	0.064	0.880	0.382	0.012	0.040	0.303	0.763	**0.241**	**0.051**	**4.706**	**0.000**
**HS 2**	**0.178**	**0.077**	**2.317**	**0.023**	**0.145**	**0.047**	**3.080**	**0.003**	0.102	0.061	1.670	0.098
**HS 3**	0.165	0.089	1.847	0.068	0.027	0.051	0.527	0.600	**0.297**	**0.071**	**4.183**	**0.000**
HS 4	0.172	0.092	1.872	0.065	0.000	0.053	−0.005	0.996	0.001	0.073	0.010	0.992
**HS 5**	**−0.288**	**0.100**	**−2.880**	**0.005**	**−0.216**	**0.058**	**−3.722**	**0.000**	0.017	0.080	0.212	0.833
HS 6	0.060	0.106	0.567	0.572	0.022	0.061	0.362	0.718	0.037	0.084	0.438	0.662

**Table 5:** Summary, units, and sources of the trait used.

**Traits**	**Units**	**Mean**	**Median (Min-Max)**	**Source**
*Quantitative traits*				
**Specific Leaf Area (SLA)**	cm^2^/g	28.1	27.4 (10.9–53.7)	[50]
**Maximum Plant Height**	cm	101.3	80 (20–500)	[51]
**Seed Weight**	g	3.1	0.8 (0.05–39.9)	[52]
**Flowering Onset**	month	5.3	6 (1–8)	[51]
**Flowering Duration**	month	4.9	4 (1–12)	[51]
**Fecundity**	average number of seeds per plant	5972	4000 (30–40,000)	[53]
**Seed Longevity**	year	33.7	26 (3–100)	[53]
**Ellenberg-L**		7.1	7 (5–9)	[54]
**Ellenberg-N**		6.5	7 (1–9)	[54]
**Ellenberg-T**		6.7	7 (5–9)	[54]
**Sensitivity to Herbicides ^1^**		4.0	4.2 (1.5–6)	[49]
*Qualitative traits*	**Modalities**	**N. of Species**		
**Life Form**	Geophytes	10		[54]
	Hemicryptophytes	12		
	Therophytes	73		
**Emergence Period**	All-year-round	29		[55]
	Autumn, Winter & Spring	15		
	Spring	9		
	Spring & Summer	24		
	Summer	18		
**Means of Dispersal**	Animal	25		[54]
	Gravity	33		
	Wind	37		

^1^ A nine-level scale (1–9) summarizes the percentage of weed control achieved with each herbicide for each weed species, based on numerous herbicide trials, with 1 indicating a low efficiency (less than 70% control) and 9 indicating a high efficiency (more than 95% control). Herbicides sensitivity is the mean value of this nine-level scale of weed control for all herbicides registered for maize in France during the 2000s.

## Appendix A

**Table A1:** Full list of species observed during the 2000s survey by decreasing order of frequency of occurrence.

*Chenopodium album* L., 1753
*Solanum nigrum* L., 1753
*Echinochloa crus-galli* (L.) P.Beauv., 1812
*Persicaria maculosa* Gray, 1821 + *Persicaria lapathifolia* (L.) Delarbre, 1800
*Amaranthus retroflexus* L., 1753
*Mercurialis annua* L., 1753
*Digitaria sanguinalis* (L.) Scop., 1771
*Polygonum aviculare* L., 1753
*Fallopia convolvulus* (L.) Á.Löve, 1970
*Calystegia sepium* (L.) R.Br., 1810
*Lysimachia arvensis* (L.) U.Manns & Anderb., 2009
*Stellaria media* (L.) Vill., 1789
*Convolvulus arvensis* L., 1753
*Senecio vulgaris* L., 1753
*Sonchus asper* (L.) Hill, 1769
*Setaria pumila* (Poir.) Roem. & Schult., 1817
*Matricaria chamomilla* L., 1753 + *Tripleurospermum inodorum* (L.) Sch.Bip., 1844
*Poa annua* L., 1753
*Setaria verticillata* (L.) P.Beauv., 1812
*Cynodon dactylon* (L.) Pers., 1805
*Atriplex patula* L., 1753
*Setaria italica* subsp. *viridis* (L.) Thell., 1912
*Capsella bursa-pastoris* (L.) Medik., 1792
*Veronica persica* Poir., 1808
*Lolium multiflorum* Lam., 1779
*Datura stramonium* L., 1753
*Lipandra polysperma* (L.) S.Fuentes, Uotila & Borsch, 2012
*Equisetum arvense* L., 1753
*Kickxia spuria* (L.) Dumort., 1827
*Panicum miliaceum* L., 1753
*Cirsium arvense* (L.) Scop., 1772
*Fumaria officinalis* L., 1753
*Panicum dichotomiflorum* Michx., 1803
*Sonchus oleraceus* L., 1753
*Alopecurus myosuroides* Huds., 1762
*Galium aparine* L., 1753
*Viola arvensis* Murray, 1770
*Sinapis arvensis* L., 1753
*Lamium purpureum* L., 1753
*Portulaca oleracea* L., 1753
*Kickxia elatine* (L.) Dumort., 1827
*Raphanus raphanistrum* L., 1753
*Plantago major* L., 1753
*Elytrigia repens* (L.) Desv. ex Nevski, 1934
*Sonchus arvensis* L., 1753
*Chenopodiastrum hybridum* (L.) S.Fuentes, Uotila & Borsch, 2012
*Sorghum halepense* (L.) Pers., 1805
*Ambrosia artemisiifolia* L., 1753
*Artemisia vulgaris* L., 1753
*Euphorbia helioscopia* L., 1753
*Rubus fruticosus* L., 1753
*Aethusa cynapium* L., 1753
*Geranium dissectum* L., 1755
*Cerastium glomeratum* Thuill., 1799
*Rumex obtusifolius* L., 1753
*Brassica nigra* (L.) W.D.J.Koch, 1833
*Agrostis stolonifera* L., 1753
*Taraxacum officinale* F.H.Wigg., 1780
*Solanum tuberosum* L., 1753
*Lysimachia foemina* (Mill.) U.Manns & Anderb., 2009
*Rumex crispus* L., 1753
*Lapsana communis* L., 1753
*Daucus carota* L., 1753
*Abutilon theophrasti* Medik., 1787
*Veronica hederifolia* L., 1753
*Helianthus annuus* L., 1753
*Brassica napus* L., 1753
*Anthemis arvensis* L., 1753
*Erigeron canadensis* L., 1753
*Ranunculus sardous* Crantz, 1763
*Veronica arvensis* L., 1753
*Trifolium arvense* L., 1753
*Lactuca serriola* L., 1756
*Cardamine hirsuta* L., 1753
*Panicum capillare* L., 1753
*Oxalis fontana* Bunge, 1835
*Spergula arvensis* L., 1753
*Digitaria ischaemum* (Schreb.) Mühl., 1817
*Paspalum dilatatum* Poir., 1804
*Reseda phyteuma* L., 1753
*Galinsoga quadriradiata* Ruiz & Pav., 1798 + *Galinsoga parviflora* Cav., 1795
*Avena fatua* L., 1753
*Chenopodiastrum murale* (L.) S.Fuentes, Uotila & Borsch, 2012
*Bidens tripartita* L., 1753
*Ranunculus repens* L., 1753
*Matricaria discoidea* DC., 1838
*Papaver rhoeas* L., 1753
*Phytolacca americana* L., 1753
*Beta vulgaris* L., 1753
*Lamium amplexicaule* L., 1753
*Avena sativa* L., 1753
*Persicaria hydropiper* (L.) Spach, 1841
*Thlaspi arvense* L., 1753
*Cyanus segetum* Hill, 1762
*Arrhenatherum elatius* subsp. *bulbosum* (Willd.) Schübl. & G.Martens, 1834
*Poa trivialis* L., 1753
*Glebionis segetum* (L.) Fourr., 1869
*Picris hieracioides* L., 1753
*Sherardia arvensis* L., 1753
*Ranunculus arvensis* L., 1753
*Malva sylvestris* L., 1753
*Chaenorrhinum minus* (L.) Lange, 1870
*Trifolium repens* L., 1753
*Plantago lanceolata* L., 1753
*Ammi majus* L., 1753
*Potentilla reptans* L., 1753
*Hordeum vulgare* L., 1753
*Trifolium pratense* L., 1753
*Sicyos angulata* L., 1753
*Geranium rotundifolium* L., 1753
*Reseda luteola* L., 1753
*Stachys arvensis* (L.) L., 1763
*Juncus bufonius* L., 1753
*Heliotropium europaeum* L., 1753
*Phragmites australis* (Cav.) Trin. ex Steud., 1840
*Euphorbia segetalis* L., 1753
*Bromus catharticus* Vahl, 1791
*Lepidium squamatum* Forssk., 1775
*Aphanes arvensis* L., 1753
*Symphytum officinale* L., 1753
*Urtica urens* L., 1753
*Carex hirta* L., 1753
*Triticum aestivum* L., 1753
*Helminthotheca echioides* (L.) Holub, 1973
*Anthemis cotula* L., 1753
*Rumex acetosa* L., 1753
*Verbena officinalis* L., 1753
*Myosotis arvensis* (L.) Hill, 1764
*Erodium cicutarium* (L.) L’Hér., 1789
*Montia fontana* L., 1753
*Lotus corniculatus* L., 1753
*Rumex acetosella* L., 1753
*Cyperus esculentus* L., 1753
*Euphorbia peplus* L., 1753
*Phalaris paradoxa* L., 1763
*Crepis foetida* L., 1753
*Stachys annua* (L.) L., 1763
*Galeopsis tetrahit* L., 1753
*Sorghum bicolor* (L.) Moench, 1794
*Vicia hirsuta* (L.) Gray, 1821
*Silybum marianum* (L.) Gaertn., 1791
*Sisymbrium officinale* (L.) Scop., 1772
*Agrostis capillaris* L., 1753
*Medicago lupulina* L., 1753
*Teesdalia nudicaulis* (L.) R.Br., 1812
*Oxalis corniculata* L., 1753
*Scandix pecten-veneris* L., 1753
*Crepis sancta* (L.) Bornm., 1913
*Vicia sativa* L., 1753
*Caucalis platycarpos* L., 1753
*Hypericum perforatum* L., 1753
*Lythrum hyssopifolia* L., 1753
*Gnaphalium uliginosum* L., 1753
*Apera spica-venti* (L.) P.Beauv., 1812
*Euphorbia exigua* L., 1753
*Geranium molle* L., 1753
*Silene latifolia* subsp. *alba* (Mill.) Greuter & Burdet, 1982
*Equisetum telmateia* Ehrh., 1783
*Torilis arvensis* (Huds.) Link, 1821
*Geranium robertianum* L., 1753
*Arenaria serpyllifolia* L., 1753
*Geranium columbinum* L., 1753
x *Triticosecale rimpaui* (M.Graebn.) Wittm. ex A.W.Hill, 1933
*Lycopsis arvensis* L., 1753
*Dactylis glomerata* L., 1753
*Misopates orontium* (L.) Raf., 1840
*Salix* L., 1753
*Trifolium campestre* Schreb., 1804
*Arabidopsis thaliana* (L.) Heynh., 1842
*Holcus mollis* L., 1759
*Ornithopus compressus* L., 1753
*Raphanus sativus* L., 1753
*Coincya monensis* subsp. *cheiranthos* (Vill.) Aedo, Leadlay & Muñoz Garm., 1993
*Fagopyrum esculentum* Moench, 1794
*Veronica serpyllifolia* L., 1753
*Sambucus nigra* L., 1753
*Erigeron sumatrensis* Retz., 1810
